# A path planning approach for ship collision avoidance integrating BRB reasoning and velocity obstacle algorithm

**DOI:** 10.1371/journal.pone.0349943

**Published:** 2026-06-17

**Authors:** Minmin Zheng, Shuwu Wang, Haiyang Tu, Qianyu Lu

**Affiliations:** 1 Shanghai Ship & Shipping Research Institute Co., Ltd., Shanghai, China; 2 College of Navigation, Jimei University, Xiamen, China; Qingdao University, CHINA

## Abstract

Reliable path planning for ship collision avoidance remains challenging in complex maritime environments with dense traffic. This study proposes a novel approach that integrates Belief-Rule-Based (BRB) reasoning with the Velocity Obstacle (VO) algorithm for multi-ship encounter scenarios. First, the BRB framework groups ships with similar movement trends and close proximity, reducing both the number of avoidance targets and decision-making complexity. Based on the grouping results, the safety domain radius for individual ships and ship groups is set proportionally to ship length. Subsequently, the VO algorithm generates safe and feasible avoidance trajectories toward the goal. The method was tested in two scenarios, showing improved navigational safety and operational efficiency in congested waters. This work provides a foundation for intelligent decision-making in maritime safety management and supports sustainable development in the shipping industry.

## 1. Introduction

As global shipping activities continue to grow, ensuring the safety of maritime navigation has become an increasingly urgent challenge. The rising vessel traffic density, particularly in busy waterways like the Taiwan Strait, exacerbates the complexity of collision avoidance in multi-ship scenarios. Traditional path planning methods often struggle to cope with these complexities, leading to increased risks of maritime accidents [1].

Recent advancements, especially the widespread adoption of the Automatic Identification System (AIS), have significantly improved the ability to track and monitor ship movements [2]. However, the large volume of AIS data generated often overwhelms maritime operators, complicating real-time decision-making. This highlights the need for more effective collision avoidance strategies that can manage the vast amount of data and enhance situational awareness in congested maritime environments.

In coastal waters, a key challenge is the dense concentration of trawl fishing vessels operating in confined areas with similar movement patterns [3]. Under such conditions, conventional collision-avoidance methods may generate avoidance paths that still intersect active fishing grounds, leaving residual collision risks. Therefore, collision-avoidance approaches that explicitly account for the operational dynamics and constrained maneuverability of fishing vessels are required to ensure safe navigation and mitigate collision risks.

To address this challenge, this study proposes a novel integrated path planning approach that combines Belief-Rule-Based (BRB) reasoning [4] with the Velocity Obstacle (VO) algorithm. The BRB reasoning framework is employed to group ships with similar movement trends and close proximity, thus reducing both the number of avoidance targets and the complexity of maneuvering decisions. The VO algorithm is then applied to generate safe and feasible avoidance paths for individual ships or ship groups, accounting for real-world maritime constraints such as vessel dynamics, environmental conditions, and encounter scenarios.

The primary objective of this research is to develop a robust and adaptive framework for multi-ship collision avoidance that leverages both BRB reasoning and the VO algorithm for path planning. By integrating these two methodologies, the proposed approach aims to reduce navigational complexity, improve operational safety, and enhance decision-making in dynamic maritime traffic conditions.

The remainder of this paper is organized as follows. Section 2 reviews existing situational awareness methods and collision avoidance algorithms relevant to maritime navigation. Section 3 presents the detailed structure and workflow of the proposed integrated approach. Section 4 describes the case studies and simulation experiments conducted to validate the effectiveness of the method. Finally, Section 5 concludes the paper and discusses potential directions for future research in intelligent maritime collision avoidance. This study contributes to the advancement of intelligent maritime traffic management by providing practical insights into improving safety in high-density shipping lanes.

## 2. Research status and gap analysis

The effectiveness of path planning for multi-ship collision avoidance is closely related to situational awareness (SA) techniques and collision avoidance strategies. Therefore, this section presents a comprehensive review and analysis of existing SA methods and collision avoidance algorithms applicable to maritime navigation. Subsequently, path planning techniques for multi-ship collision avoidance are introduced in detail.

### 2.1. Situational awareness methods

Situational awareness in maritime navigation is defined as the perception of the environment, its comprehension, and the projection of its future status. Traditional situational awareness (SA) methods in maritime navigation have heavily relied on visual cues and manual tracking of vessels, both of which are labor-intensive and prone to human error. With the widespread adoption of Automatic Identification System (AIS) technology, real-time tracking and monitoring of vessel movements have significantly improved.

Several models have been proposed to enhance SA in maritime contexts, leveraging real-time AIS data for better decision-making. For instance, Tian et al. [5] proposed a method that enhances SA by identifying pairwise ship encounters using AIS data, extracting valuable knowledge from different encounter situations. Similarly, Huang et al. [6] developed a regional collision risk assessment framework that uses a directed distance-based DBSCAN algorithm, which helps measure collision risks in complex waterways.

**Data fusion techniques** have played a key role in improving SA. By integrating AIS data with radar and environmental sensor data, a more accurate and comprehensive representation of the maritime environment can be constructed. Techniques such as Kalman filtering and Bayesian networks have been applied for vessel tracking and uncertainty reduction in data interpretation [5,6].

Furthermore, **cognitive models** have emerged as important tools for decision-making under uncertainty. These models, which account for human cognitive limitations and biases, aim to improve SA by aligning with the cognitive abilities of navigators in high-traffic or complex scenarios [7,8]. Gao et al. [7] proposed a deep learning-based framework for regional collision risk assessment using Transformer networks, while Yang et al. [8] introduced a composite distance measure and constrained Shared Nearest Neighbor (SNN) clustering to identify high-risk multi-ship encounters in complex waters.

### 2.2. Collision avoidance algorithms

Collision avoidance algorithms are crucial in ensuring safe navigation by enabling vessels to maneuver safely in congested or high-risk areas. These algorithms can generally be classified into two major categories: rule-based systems and optimization-based approaches.

**Rule-based systems** typically apply established maritime navigation rules, such as the International Regulations for Preventing Collisions at Sea (COLREGs), to determine safe maneuvers. These systems use heuristic methods to predict potential collisions based on vessel relative positions, velocities, and trajectories. While effective in many standard scenarios, rule-based systems may struggle in complex multi-ship encounters, where strict rule adherence may be insufficient [9,10]. For example, Tong et al. [9] proposed an approach for identifying influential ships in multi-ship encounter situations using a complex network of ship encounter clusters, and Liu et al. [10] introduced dynamic conflict cluster detection to improve decision-making in such contexts.

**Optimization-based approaches** employ mathematical optimization methods to identify the best course of action for a vessel, minimizing collision risks while considering operational constraints like fuel efficiency and time delays. Methods such as Dynamic Programming and Genetic Algorithms have been widely used in maritime path planning and collision avoidance [11,12]. Guan et al. [11] proposed a generalized behavior decision-making model for ship collision avoidance that incorporates reinforcement learning (RL) and COLREGs. Meanwhile, Zhen et al. [12] introduced a regional ship collision risk assessment method that considers encounter density in multi-ship situations, aiming to enhance maritime traffic safety.111

In recent years, Artificial Intelligence (AI) and Machine Learning (ML) have gained increasing attention for their ability to adapt to dynamic environments and improve collision avoidance strategies. AI-based systems can learn from past encounters, enabling predictive modeling for future decision-making. Pan et al. [13] proposed a deep reinforcement learning-based multi-ship collision avoidance decision-making model that considers inter-ship relationships, safety, efficiency, and COLREGs compliance. Fan et al. [14] further developed a progressive deep reinforcement learning approach for intelligent collision avoidance in unmanned surface vehicles, improving the adaptability of learning-based methods in complex environments.

### 2.3. Path planning and collision avoidance in multi-ship scenarios

With the growing complexity of maritime traffic, effective path planning and collision avoidance are critical for autonomous ships. Recent research has integrated AI and optimization algorithms to address these challenges, enhancing both safety and operational efficiency.

Ali et al. [15] introduced Safety-enhanced Path Planning (SPP), combining A* with local navigation strategies to navigate static and dynamic obstacles. Similarly, Liu et al. [16] proposed a COLREGs-compliant autonomous collision avoidance and adaptive LOS-based motion control method for unmanned surface vehicles in complex waters. Both approaches support safer navigation under dynamic obstacles and operational constraints.

Prediction-based path planning has also gained attention. Zhu et al. [17] developed a method that uses Convolutional Neural Networks (CNN) and Dynamic Time Warping (DTW) to predict future ship positions and improve decision-making during multi-ship encounters. This method outperforms traditional A* in terms of safety and efficiency. Xiang et al. [18] applied Improved Particle Swarm Optimization (IPSO) to optimize ship maneuvering, balancing collision risk, energy use, and steering costs.

Collaborative collision avoidance is essential when multiple ships interact. Zhen et al. [19] proposed a deterministic search algorithm for multi-ship scenarios, integrating safety and economic factors and ensuring compliance with COLREGs. Zhang et al. [20] further proposed an efficient collaborative collision avoidance algorithm that integrates COLREGs, ship maneuverability, and a velocity-obstacle-based method for complex multi-ship encounter scenarios.

Finally, real-world validation is crucial. He et al. [21] proposed a dynamic domain-based system for autonomous ships, using virtual potential fields to generate safe paths and conduct real-world tests in coastal waters, showing effective collision avoidance even with dynamic obstacles.

Overall, recent studies show a clear shift from single-vessel path generation to multi-ship interaction modelling, COLREGs-aware decision-making, and learning-based cooperative avoidance. For example, recent work has incorporated practical decision-making requirements into intelligent collision avoidance strategies [22] and used proximal policy optimization to improve COLREGs-compliant multi-ship avoidance [23]. However, these methods generally focus on direct decision policy learning or individual-vessel interaction modelling. In dense traffic, the number of target ships can still increase the decision dimension and may produce overly conservative maneuvers. This limitation provides the motivation for combining BRB-based ship grouping with VO-based path planning in this study.

### 2.4. Research gaps and motivation

While significant progress has been made in ship collision avoidance, several challenges remain. One major issue is data overload: if all AIS records and all surrounding ships are considered directly during collision avoidance, the decision space expands rapidly and may delay real-time response. Advanced methods for dynamic risk assessment, interaction modelling, filtering, grouping, and prioritizing critical targets are therefore needed [22,24].

Another challenge is the integration of situational awareness (SA) and collision avoidance. Many existing systems still treat traffic perception, risk identification, and avoidance decision-making as separate processes, which may lead to fragmented decisions in multi-ship encounters. Combining these elements within a unified framework can improve both the accuracy and efficiency of collision avoidance systems [22].

Finally, real-time decision support remains a key challenge. Although recent learning-based methods have improved autonomous decision-making in multi-ship encounters [23], practical navigation still requires interpretable and computationally efficient guidance that can be generated quickly from dynamic AIS information.

To address these challenges, this study proposes an integrated approach that combines BRB reasoning with the VO algorithm to enhance both situational awareness and path planning for multi-ship collision avoidance. Unlike learning-based collision-avoidance approaches that directly learn individual maneuvering policies [14] or collaborative collision-avoidance approaches that still account for every ship in the scene [20], this study introduces a new BRB–VO integrated framework. In this framework, the BRB-based dynamic grouping reduces the decision dimensionality by clustering vessels with similar navigational trends, while the VO algorithm constructs the corresponding safety domains and generates feasible avoidance trajectories. This integration prevents the overly conservative paths that often result from treating dense traffic as independent targets and enhances adaptability in highly dynamic maritime environments. By grouping ships with similar navigation trends, the BRB framework reduces the number of avoidance targets, simplifying decision-making. The VO algorithm then generates feasible and safe collision avoidance paths for each ship group, accounting for dynamic encounter situations and maritime constraints.

## 3. A proposed approach

This section presents an integrated multi-ship collision avoidance path planning approach that combines Belief-Rule-Based (BRB) reasoning with the Velocity Obstacle (VO) algorithm. The method utilizes AIS data to dynamically group ships exhibiting similar movement trends, thereby reducing the complexity of encounter situations. Based on the grouping results, the path planning module subsequently generates collision-free trajectories that comply with navigational safety constraints, enhancing both safety and decision-making efficiency in congested maritime environments.

### 3.1. Overall workflow of the approach

**Fig 1:**
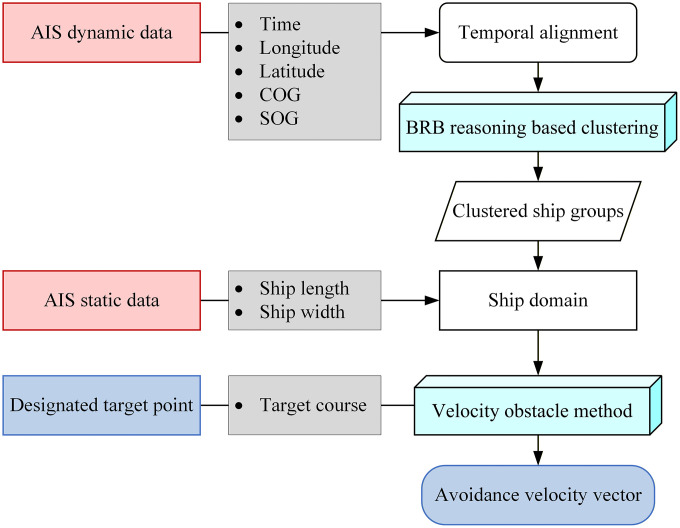
The overall workflow of the proposed path planning approach.

Fig 1 shows the workflow of the proposed path planning method, which begins with the preprocessing of AIS dynamic data to achieve temporal alignment. This is accomplished by interpolating and resampling ship position (longitude, latitude), COG (Course Over Ground), SOG (Speed Over Ground), and time records, ensuring a consistent time base for all ships in the encounter scenario.

Once aligned, the BRB reasoning framework is applied to analyze ship movement patterns and spatial relationships, grouping ships with similar navigation trends and close proximity. This reduces the number of targets and simplifies subsequent decision-making. The safety domain for each ship or ship group is then determined according to its principal dimensions obtained from AIS static information, providing adaptive separation constraints.

Using these inputs, the Velocity Obstacle algorithm generates collision-free velocity vectors that guide the Own Ship (OS) toward the designated goal point while satisfying the safety domain requirements. The resulting sequence of velocity vectors forms a navigable and safe avoidance path, which can be applied in real time or used for simulation-based evaluations.

### 3.2. AIS data temporal alignment

The temporal alignment of AIS data is a critical process for ensuring the consistency and reliability of ship trajectory analysis. AIS dynamic data, which includes temporal-spatial parameters such as timestamp, longitude, latitude, COG, and SOG, are inherently asynchronous due to variations in data transmission intervals and potential signal reception delays. To mitigate these issues, the temporal alignment module standardizes the AIS data streams from all ships within the operational area, ensuring synchronized time frames. This alignment facilitates precise and unified analysis of multi-ship interactions, thereby improving the accuracy of subsequent collision avoidance decision-making.

The temporal alignment process involves the following key steps:

1) Data Collection: Raw AIS dynamic data are acquired from multiple ships operating within the designated area. These data are inherently asynchronous due to variations in transmission frequencies and potential signal delays, resulting in irregular timestamps.
2) Time Reference Selection: A uniform time reference is established, such as the system clock or a predefined sampling rate, to which all ship data are synchronized.
3) Interpolation/Resampling: For each ship, the AIS data are interpolated or resampled to match the common time reference. Linear interpolation is utilized due to its simplicity and computational efficiency, ensuring consistent temporal alignment across all ships.

The interpolated value of a parameter P (e.g., longitude, latitude, COG, or SOG) at time $t_{ref}$ is calculated as:


$$\begin{array}{c} P\left(t_{ref}\right)=P\left(t_{i}\right)+\frac{P\left(t_{i+\text{1}}\right)-P\left(t_{i}\right)}{t_{i+\text{1}}-t_{i}}\cdot \left(t_{ref}-t_{i}\right) \end{array}$$ (1)

where $P\left(t_{i}\right)$ and $P\left(t_{i+\text{1}}\right)$ are the parameter values at timestamps $t_{i}$ and $t_{i+\text{1}}$, respectively. $t_{ref}$ lies between $t_{i}$ and $t_{i+\text{1}}$.

The output of the temporal alignment process is a set of synchronized AIS dynamic data for all ships, which are subsequently utilized for clustering and collision risk analysis.

### 3.3. BRB reasoning-based clustering

The BRB reasoning-based clustering module is designed to group ships based on their movement patterns and collision risk profiles. This process involves three primary steps: selecting the clustering metric, applying the BRB reasoning method, and determining the ship groups.

#### 3.3.1. Selection of clustering metric.

**Fig 2:**
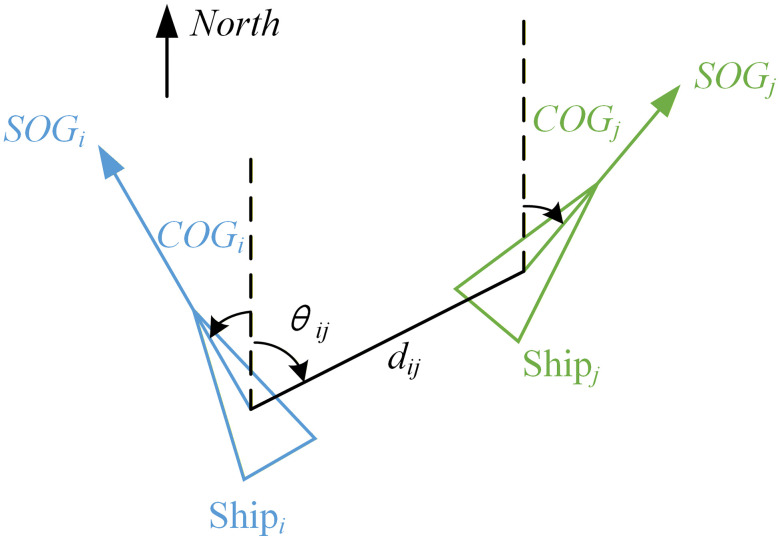
Schematic diagram of the dynamic parameters and their relationship between two ships.

The selection of the clustering metric is a pivotal step in the BRB-driven clustering process, as it defines the criteria for grouping ships according to their navigational dynamics and collision risk profiles. AIS dynamic data (e.g., latitude, longitude, COG, SOG) are employed to construct the clustering metric, enabling a comprehensive similarity measure between ships. **Fig 2** shows the relationship between two ships.

**Fig 2** shows the dynamic parameters and their relationship between ship i and ship j. Here, $SOG_{i}$ and $SOG_{j}$ represent the speed over ground of ship i and ship j, respectively. $COG_{i}$ and $COG_{j}$ denote the course over ground of ship i and ship j, respectively. $d_{ij}$ is the distance between ship i and ship j, and $\theta_{ij}$ indicates the true bearing of ship j relative to ship i.

Based on the above definition, this research establishes five metrics as clustering criteria, which are defined as follows:

1) Metric $x_{\text{1}}$: Distance describes their spatial proximity, defined as follows:
  $$\begin{array}{c} x_{1}=d_{ij} \end{array}$$ (2)
2) Metric $x_{\text{2}}$: Speed Difference, defined as follows:
  $$\begin{array}{c} x_{2}=\left|SOG_{i}-SOG_{j}\right| \end{array}$$ (3)
3) Metric $x_{\text{3}}$: Course Difference together with $x_{\text{2}}$ describe how similar the motion trends of the two vessels, defined as follows:
  $$\begin{array}{c} x_{3}=\left|COG_{i}-COG_{j}\right| \end{array}$$ (4)
4) Metric $x_{\text{4}}$: Average Speed reflects the overall speed level of the two vessels in the encounter scenario, defined as follows:
  $$\begin{array}{c} x_{4}=\frac{\left(SOG_{i}+SOG_{j}\right)}{2} \end{array}$$ (5)
5) Metric $x_{\text{5}}$: Course Bearing Angle describes their relative attitude, i.e., the geometric orientation of one vessel with respect to the other, defined as follows:
  $$\begin{array}{c} x_{5}=\left|\frac{\left(COG_{i}+COG_{j}\right)}{2}-\theta_{ij}\right| \end{array}$$ (6)

#### 3.3.2. BRB reasoning method.

1) Representation of the BRB
  Yang et al. [8] proposed an extension to the IF-THEN rule description format, where the consequent is expressed as a belief distribution, which is then implemented in the BRB system. A typical BRB system consists of multiple belief rules, with rule weights introduced to differentiate the influence of each rule on the inference results. To address the uncertainty in expert knowledge within the rule base, attribute weights are also incorporated. Therefore, the rules in the BRB system are represented as follows:
  $$\begin{array}{c} \begin{array}{c} R_{l}:\;if\;\left(x_{\text{1}}\;is\;\overset{l}{\underset{\text{1}}{A}}\right)\cap \left(x_{\text{2}}\;is\;\overset{l}{\underset{\text{2}}{A}}\right)\cap \cdots \cap \left(x_{I}\;is\;\overset{l}{\underset{I}{A}}\right),\\ \;then\;\{\left(D_{\text{1}},\beta_{\text{1},\;l}\right),\left(D_{\text{2}},\beta_{\text{2},l}\right),\cdots ,(D_{M},\beta_{M,l})\}\\ \sum_{m=\text{1}}^{M}\beta_{m,l}\leq \text{1} \end{array} \end{array}$$ (7)
  where $x_{i}\;(i=\text{1},\text{2},\ldots ,I)$ represents the feature of the $l_{th}$ rule, and $\overset{l}{\underset{i}{A}}$ (i=1,2,…,I;l=1,2,…,L) denotes the reference value corresponding to the $i_{th}$ feature $x_{i}$ of the $l_{th}$ rule. I is the number of features, and L is the total number of rules in the rule base. $D_{m}$ (m=1,2,…,M) represents the consequent attribute of the belief rule, and $\beta_{m,l}$ (m=1,2,…,M;l=1,2,…,L) denotes the belief distribution corresponding to the consequent attribute $D_{m}$. M is the number of consequent attributes.
  In this paper, the rule weight of the $l_{th}$ rule is denoted by $\theta_{l}$ (l=1,2,…,L), which reflects the relative importance of this rule in comparison to other belief rules within the BRB model’s inference process. The attribute weight of the feature is denoted by $\delta_{i}$ (i=1,2,…,I), representing the relative importance of the $i_{th}$ feature in relation to the other features. In the initial rule base, the output results and their corresponding belief distribution values for each belief rule are provided either by experienced experts or derived through statistical analysis of historical data. Initially, both feature attribute weights and rule weights are set to 1, indicating equal importance and influence.
2) Calculation of Matching Degree
  The information processed by the BRB system is complex and diverse, with varying units and physical interpretations. Yang et al. [8] proposed a method to transform this basic information into a belief distribution structure. According to this approach, the matching degree between the antecedent feature and the reference value can be used to construct the belief structure. Let $\overset{l}{\underset{i,j}{\alpha }}$ represent the matching degree between the input feature $x_{i}$ and the $j_{th}$ reference value $\overset{l}{\underset{i,j}{A}}$ in the $l_{th}$ rule. The calculation method for this matching degree is given by the following equation:
  $$\begin{array}{c} \overset{l}{\underset{i,j}{\alpha }}=\frac{\overset{l}{\underset{i,j+\text{1}}{A}}-x_{i}}{\overset{l}{\underset{i,j+\text{1}}{A}}-\overset{l}{\underset{i,j}{A}}},\overset{l}{\underset{i,j}{A}}\leq x_{i}\leq \overset{l}{\underset{i,j+\text{1}}{A}} \end{array}$$ (8)
  $$\begin{array}{c} \overset{l}{\underset{i,j+\text{1}}{\alpha }}=\frac{x_{i}-\overset{l}{\underset{i,j}{A}}}{\overset{l}{\underset{i,j+\text{1}}{A}}-\overset{l}{\underset{i,j}{A}}},\overset{l}{\underset{i,j}{A}}\leq x_{i}\leq \overset{l}{\underset{i,j+\text{1}}{A}} \end{array}$$ (9)
  where $\overset{l}{\underset{i,j}{A}}$ (i=1,2,…,I;j=1,2,…,J−1) represents the $j_{th}$ reference value of the $i_{th}$ input feature under the $l_{th}$ rule. J denotes the number of reference levels for the $i_{th}$ input feature. When the value of the feature $x_{i}$ lies between two reference values, the matching degrees of $x_{i}$ to these two reference values can be calculated using Equations (8) and (9), while the matching degrees to other reference values are assigned as 0. When $x_{i}\leq \overset{l}{\underset{i,\text{1}}{A}}$ or $x_{i}\geq \overset{l}{\underset{i,J}{A}}$, the matching degrees of $x_{i}$ to $\overset{l}{\underset{i,\text{1}}{A}}$ and $\overset{l}{\underset{i,J}{A}}$ are both set to 1, while the matching degrees to all other reference values are set to 0.
3) Rule-Based Reasoning
  The inference process of the BRB system consists primarily of two steps: rule activation and the fusion of activated rules.
  Step one: rule activation. The activation weight of a rule represents the degree to which it influences the inference results of the BRB system. In this paper, the closer the feature information of the input data x is to the feature combination of a specific rule, the greater the activation weight of that rule. If $w_{l}=\text{0}$, it indicates that the rule will not be activated. The activation weight $w_{l}$ of the $l_{th}$ rule is calculated as follows:
  $$\begin{array}{c} w_{l}=\frac{\theta_{l}\times \prod_{i=\text{1}}^{I}{\left(\overset{l}{\underset{i,j}{\alpha }}\right)}^{\overset{l}{\underset{i}{\overset{―}{\delta }}}}}{\sum_{l=\text{1}}^{L}[\theta_{l}\times \prod_{i=\text{1}}^{I}{\left(\overset{l}{\underset{i,j}{\alpha }}\right)}^{\overset{l}{\underset{i}{\overset{―}{\delta }}}}]} \end{array}$$ (10)
  where $\prod_{i=\text{1}}^{I}{\left(\overset{l}{\underset{i,j}{\alpha }}\right)}^{\overset{l}{\underset{i}{\overset{―}{\delta }}}}$ represents the matching degree between the input data feature x and the entire feature combination of the $l_{th}$ rule. Here, $\overset{l}{\underset{i}{\overset{―}{\delta }}}=\overset{l}{\underset{i}{\delta }}/\underset{i=\text{1},\text{2},\cdots I}{max}\left(\overset{l}{\underset{i}{\delta }}\right)$ denotes the relative weight of the feature, and $\theta_{l}$ is the rule weight, as previously defined.
  Step two: fusion of the activated rules. Using the Evidential Reasoning (ER) algorithm, the activated rules are fused to calculate the predicted belief degree corresponding to each evaluation result. The calculation method is provided in the following equations:
  $$\begin{array}{c} \beta_{m}=\frac{\mu \times [\prod_{l=\text{1}}^{L}(w_{l}\beta_{m,l}+\text{1}-w_{l}\sum_{m=\text{1}}^{M}\beta_{m,l})-\prod_{l=\text{1}}^{L}(\text{1}-w_{l}\sum_{m=\text{1}}^{M}\beta_{m,l})]}{\text{1}-\mu \times [\prod_{l=\text{1}}^{L}\left(\text{1}-w_{l})\right]}(m=\text{1},\text{2},\cdots ,M) \end{array}$$ (11)
  $$\begin{array}{c} \mu ={\left[\sum_{m=\text{1}}^{M}\prod_{l=\text{1}}^{L}(w_{l}\beta_{m,\;l}+\text{1}-w_{l}\sum_{m=\text{1}}^{M}\beta_{m,\;l})-(M-\text{1})\times \prod_{l=\text{1}}^{L}(\text{1}-w_{l}\sum_{m=\text{1}}^{M}\beta_{m,l})\right]}^{-\text{1}} \end{array}$$ (12)
  where $\beta_{m}$ represents the belief degree of the $m_{th}$ evaluation result calculated using the ER algorithm, and $\beta_{m,l}$ denotes the belief distribution of the $m_{th}$ evaluation result in the $l_{th}$ rule. The final inference result D of the BRB model can be expressed as follows:
  $$\begin{array}{c} D=\left\{\left(D_{m},\beta_{m}\right),m=\text{1},\text{2},\cdots ,M\right\} \end{array}$$ (13)
  The algorithm is implemented in a local manner: for each own ship, only vessels within a predefined interaction radius are considered. Let K be the number of such neighboring ships. In each planning step, the main computational cost scales approximately linearly with K, rather than with the total number of ships in the area. Thus, even in high-density traffic, the computational load for each ship is bounded by the local interaction radius and the corresponding number of effective neighbors.

#### 3.3.3. Determination of ship group.

Based on BRB reasoning, ship group determination follows a transitive relationship. Specifically, if ship i and ship j are classified into the same group, and ship j and ship k are also classified into the same group, then ships i, j, and k are grouped together. This transitive property ensures consistency and logical coherence in the clustering process. The BRB system evaluates the navigation characteristics and collision risk profiles of ships to determine their group affiliations, facilitating effective clustering and collision avoidance strategies.

The resulting ship clusters are then used as input entities for collision resolution. This allows the path planner to avoid treating each ship individually when appropriate, thereby reducing computational load and minimizing unnecessary path conflicts.

### 3.4. Determination of ship domain radius

The determination of the domain radius is a critical component of collision avoidance and navigation safety, as it defines the spatial boundary within which a vessel or a group of vessels operates. This section is divided into two parts: Domain Radius of a Single Ship and Domain Radius of a Ship Group.

#### 3.4.1. Domain radius of a single ship.

Typically, the domain radius for a single ship is determined based on factors such as the vessel’s size, maneuverability, speed, and environmental conditions. This radius represents the minimum safe distance that must be maintained around the ship to prevent collisions.

For the purpose of this paper, which subsequently applies the VO algorithm for collision avoidance, a circular ship domain model is adopted for simplicity. The center of the circle is located at the current position of the ship, and the ship domain radius is defined as a multiple of the ship’s length, as described below:


$$\begin{array}{c} \overset{SD}{\underset{i}{R}}=a\cdot L_{i} \end{array}$$ (14)

where $L_{i}$ represents the length of ship i, and $\overset{SD}{\underset{i}{R}}$ denotes the ship domain radius of ship i. a is the coefficient of the ship domain range.

#### 3.4.2. Domain radius of ship group.

The determination of the domain radius for a ship group is carried out in three steps:

**Step one:** Identify the ship domains of all ships within group g and select the largest domain radius as $\overset{\text{1}}{\underset{g}{R}}$.


$$\begin{array}{c} \overset{\text{1}}{\underset{g}{R}}=max\left(\overset{SD}{\underset{i}{R}}\right)(i\in g) \end{array}$$ (15)

**Step two:** Calculate the radius $\overset{\text{2}}{\underset{g}{R}}$ and the center point of the minimum covering circle that encompasses the positions of all ships in the group, following the smallest enclosing circle formulation [25].

**Step three:** Determine the domain radius of the ship group as $\overset{SD}{\underset{g}{R}}=\overset{\text{2}}{\underset{g}{\overset{\text{1}}{\underset{g}{R}}+R}}$.

This approach ensures that the domain radius of the ship group accounts for both the largest individual ship domain and the spatial distribution of all ships within the group, providing a comprehensive safety boundary for collision avoidance.

### 3.5. VO-based collision resolution

The VO algorithm is employed in ship collision avoidance due to its effectiveness in dynamic environments. It offers a real-time, predictive approach to collision avoidance by analyzing the relative velocities and positions of vessels. The algorithm is particularly well-suited for maritime applications as it can handle multiple moving obstacles and complex navigation scenarios, ensuring safe and efficient path planning [26].

The obstacle zone radius in the VO algorithm is determined based on both the ship domain of the OS and Target Ships (TSs). For a single ship i, the obstacle zone radius is denoted as $R_{i}$, while for a ship group g, the obstacle zone radius is denoted as $R_{g}$. The ship domain radius of the OS is $\overset{SD}{\underset{OS}{R}}$. The values $R_{i}$ and $R_{g}$ are defined as follows:


$$\begin{array}{c} R_{i}=\text{max}\left(\overset{SD}{\underset{i}{\overset{SD}{\underset{OS}{R}},R}}\right) \end{array}$$ (16)

$$\begin{array}{c} R_{g}=\text{max}\left(\overset{SD}{\underset{OS}{R}},\overset{\text{1}}{\underset{g}{R}}\right)+\overset{\text{2}}{\underset{g}{R}} \end{array}$$ (17)

The velocity decision space is defined as follows: the maximum and minimum velocities are $v_{max}$ and $v_{min}$, respectively, with a velocity resolution of $v_{step}$. The maximum and minimum heading change angles are $\theta_{max}$ and $\theta_{min}$, respectively, with a heading change resolution of $\theta_{step}$.

The loss function for the VO algorithm is defined as:


$$\begin{array}{c} J=\beta \cdot \left|v_{sel}-v_{set}\right|+(\text{1}-\beta )\cdot \left|\theta_{sel}-\theta_{goal}\right| \end{array}$$ (18)

where $v_{sel}$ and $\theta_{sel}$ are the selectable velocities and courses, respectively. $v_{set}$ is the set velocity. $\theta_{goal}$ is the true bearing of the goal point relative to the current position of OS. And β∈[0,1] is the weight that balances the velocity change and the course deviation. A larger β increases the penalty on speed changes and thus favors solutions with nearly unchanged speed, whereas a smaller β increases the penalty on heading changes and thus favors solutions with nearly unchanged heading. In practice, β is selected according to the control preference of the navigator and the specific operational scenario.

The objective of the collision resolution is to select the optimal velocity $\overset{*}{\underset{sel}{v}}$ and course $\overset{*}{\underset{sel}{\theta }}$ from the velocity decision space, minimizing the loss function J defined in Equation (18).

In this study, the VO module uses the output of the BRB-driven clustering process to determine whether collision avoidance should be performed at the individual vessel level or at the group level. This ensures that the planned path does not unnecessarily cross cohesive clusters of ships moving in similar directions.

## 4. Case study and results

In order to validate the performance of the proposed multi-ship collision avoidance path planning method, this section establishes a simulation environment for testing. The simulated environment consists of two different scenarios. First, the parameter configuration is introduced, followed by an overview of the study area. The clustering results are then presented, and finally, the collision avoidance outcomes with and without BRB clustering are compared for each test.

### 4.1. Parameter configuration

The parameter settings in **Table 1** correspond to the input features of the BRB model, which is employed to cluster ships with close distance and similar movement trends. Each metric is discretized into several semantic values to capture essential differences while avoiding excessive rule complexity. Specifically, distance is divided into three levels to reflect near, medium, and far encounters, which directly indicate the likelihood of interaction. Speed difference, course difference, average speed, and course bearing angle are assigned two representative thresholds, sufficient to distinguish between similar and dissimilar motion patterns in practical navigation scenarios. These heuristic settings, derived from maritime experience and collision risk characteristics, ensure that the BRB model can effectively group ships with comparable behaviors into the same cluster, thereby reducing the dimensionality of multi-ship encounters.

**Table 1:** Sub-features and their semantic values (SV) under input features (IF).

SV IF	${𝐱}_{1}$ (nm)	${𝐱}_{2}$ (kn)	${𝐱}_{3}$ (°)	${𝐱}_{4}$ (kn)	${𝐱}_{5}$ (°)
**0**	$A_{\text{1}\text{1}}$=1/3	$A_{\text{2}\text{1}}$=2	$A_{\text{3}\text{1}}$=10	$A_{\text{4}\text{1}}$=2	$A_{\text{5}\text{1}}$=22.5
**1**	$A_{\text{1}\text{2}}$=2/3	$A_{\text{2}\text{2}}$=4	$A_{\text{3}\text{2}}$=20	$A_{\text{4}\text{2}}$=4	$A_{\text{5}\text{2}}$=45
**2**	$A_{\text{1}\text{3}}=\text{1}$	–	–	–	–

Note: IF refers to Input Features and SV refers to Semantic Value.

The BRB rules shown in **Table 2** were designed to map combinations of input features to the likelihood of two ships being clustered into the same group. The logic follows practical navigation principles: when ships are close in distance, with small differences in speed, course, and relative bearing, the probability of belonging to the same group (D1) is assigned a high value (e.g., 0.9), while the probability of belonging to different groups (D2) is low. Conversely, when the distance is large or the motion trends differ significantly, the rules assign a higher probability to D2, indicating separation into different groups. The probabilistic representation allows the model to capture the uncertainty and gradual transitions between similar and dissimilar states, rather than enforcing rigid thresholds. These rules serve as the knowledge base of the BRB model, enabling flexible clustering decisions consistent with maritime encounter scenarios.

**Table 2:** BRB reasoning-based clustering rules.

Rules	Input Features					Output features	
	${𝐱}_{1}$	${𝐱}_{2}$	${𝐱}_{3}$	${𝐱}_{4}$	${𝐱}_{5}$	${𝐃}_{1}$	${𝐃}_{2}$
**1**	0	0	0	0	0	0.9	0.1
**2**	0	0	0	0	1	0.9	0.1
**3**	0	0	0	1	0	0.9	0.1
**…**	…	…	…	…	…	…	…
**46**	2	1	1	0	1	0.1	0.9
**47**	2	1	1	1	0	0.1	0.9
**48**	2	1	1	1	1	0.1	0.9

Note: ${\text{D}}_{\text{1}}$ represents the probability that the ships belong to the same group, while ${\text{D}}_{\text{2}}$ represents the probability that the ships belong to different groups.

The parameters in **Table 3** were selected to balance safety, maneuverability, and computational efficiency. The circular ship domain coefficient a was set to 3 to provide a sufficient safety margin, while the coefficient β in Equation (18) used for VO algorithm is set to 0.9. The velocity search space was defined from 8 kn ($v_{min}$) to 12 kn ($v_{max}$) with a step size ($v_{step}$) of 0.5 kn, and the heading search range was constrained to $\left[-\theta_{max},\;\theta_{max}\right]$ with $\theta_{max}=\pi /\text{3}$ and $\theta_{step}=\pi /\text{3}\text{6}$, reflecting realistic maneuvering limits. For the experimental setup, the OS length ($L_{OS}$) was set 100 m and the initial speed ($v_{OS}$) to 10 kn, representing a typical merchant ship scenario.

**Table 3:** Other parameters used in this research.

Items	Values	Items	Values	Items	Values
a	3	$v_{min\;}$	8 kn	$\theta_{step}$	π/36
β	0.9	$v_{step}$	0.5 kn	$L_{OS}$	100 m
$v_{max}$	12 kn	$\theta_{max}$	π/3	$v_{OS}$	10 kn

$L_{OS}$ is the length of OS.

Additionally, the parameters related to the VO algorithm are listed in **Table 3**.

### 4.2. Overview of study area and clustering results

**Fig 3:**
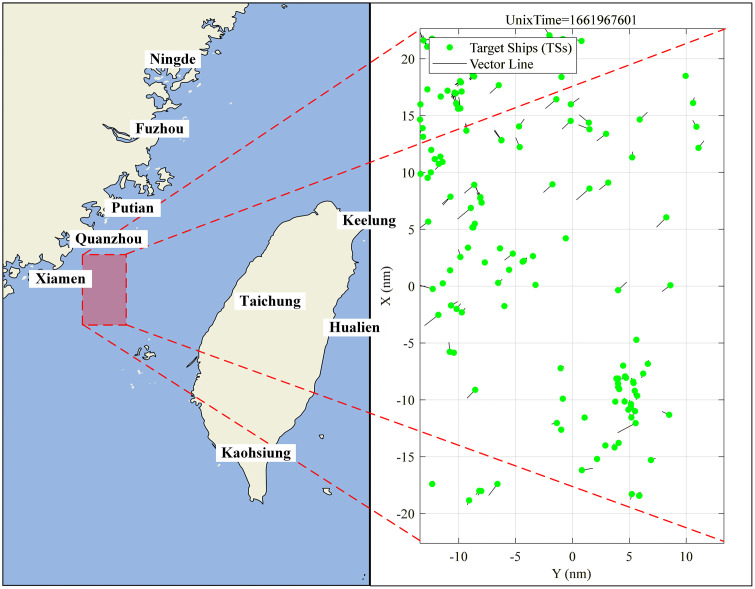
Overview of the study area and data. The map was created using Cartopy (a Python library) with base map data from Natural Earth. Natural Earth data is in the public domain (https://www.naturalearthdata.com/) and free to use in any project.

The study area for this research is located near Quanzhou in the Taiwan Strait, with the extracted AIS data covering a rectangular region of 44 nautical miles (nm) in length and 26 nm in width (see **Fig 3**). The AIS data spans the entire day of September 1, 2019, providing a comprehensive view of vessel distribution within the region. **Fig 3** shows an interpolated data frame. Green solid points denote ungrouped TSs, and black solid lines indicate each vessel’s course. This provides an intuitive basis for subsequent analysis.

**Fig 4:**
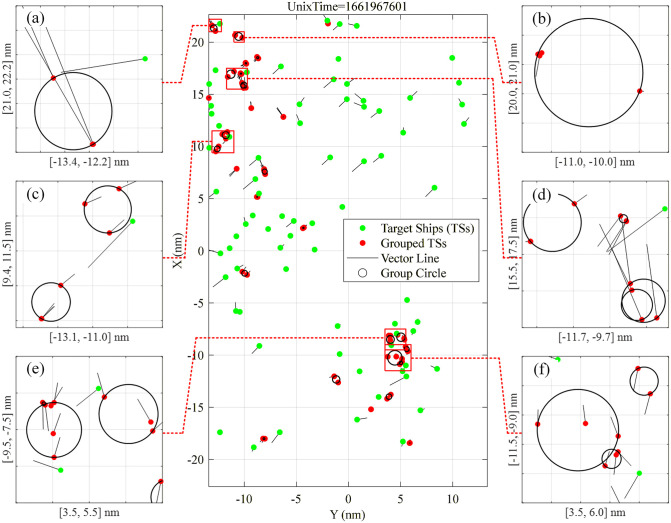
The results of BRB clustering on data in Fig 3.

Additionally, **Fig 4** shows the BRB clustering results based on the data shown in **Fig 3** and provides details of the clustering outcomes. Specifically, panels (a) and (b) depict a clustered ship group, with (a) showing the group sailing northwest by north and (b) indicating that the group is in a stopped or anchored state. Panels (c) and (e) display two separate clustered ship groups, with (c) showing both groups sailing northeast and (e) showing both groups in a stopped or anchored state. Panels (d) and (f) present additional clustered ship groups, showcasing more complex scenarios. **Fig 4** shows the red solid points represent the TSs after clustering, indicating the groups identified by the BRB method. The black circles outline the boundaries of the groups, visualizing the spatial extent of each clustered ship group.

### 4.3. Test scenarios and comparative results

To thoroughly evaluate the performance of the proposed multi-ship collision avoidance path planning algorithm, two distinct test scenarios were designed. These scenarios aim to assess the algorithm’s effectiveness in handling complex maritime traffic situations.

#### 4.3.1. Test Scenario 1.

**Fig 5:**
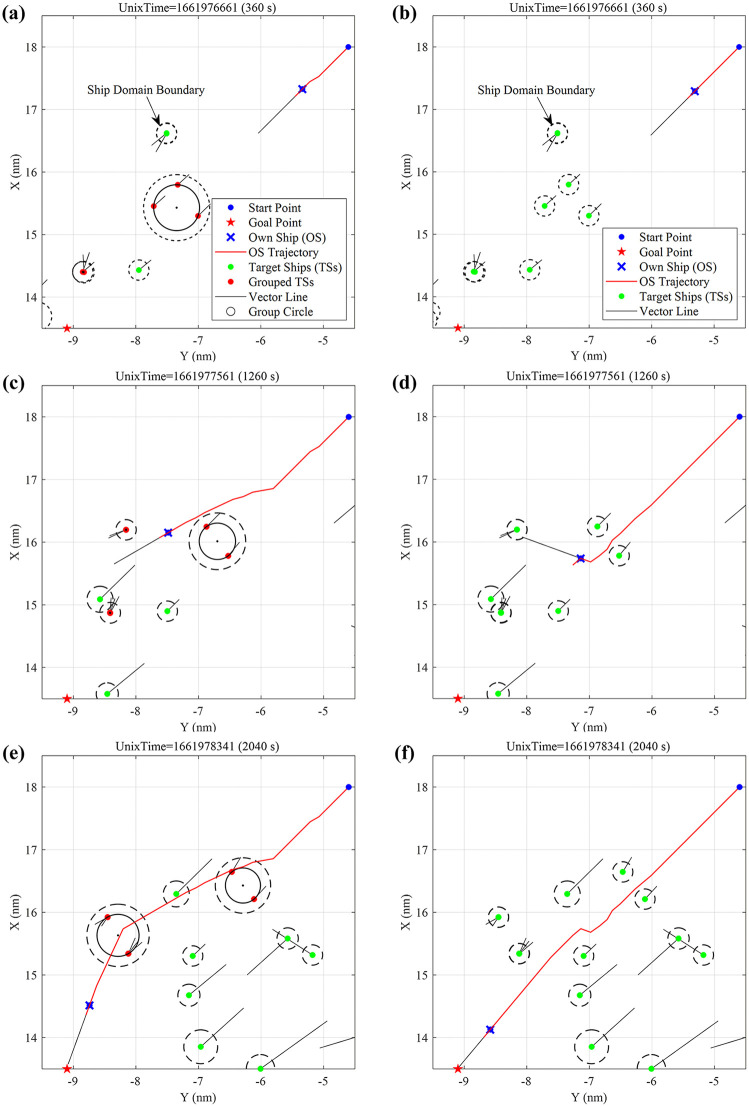
Collision avoidance path planning results for Scenario 1 with and without BRB clustering. Subfigures **(a)**, **(c)**, and **(e)** show the path planning process with BRB clustering at different stages, while subfigures **(b)**, **(d)**, and **(f)** show the corresponding results without BRB clustering.

In Test Scenario 1, the goal point is set at (13.5, −9.1) nm, with the starting position of the OS at (18, −4.6) nm and an initial course of 225 degrees. **Fig 5** shows the collision avoidance path planning results, where panels (a), (c), and (e) present the results with BRB clustering, while panels (b), (d), and (f) present the results without BRB clustering.

Various symbols are used for better understanding: the blue solid points represent the start point, while the red solid pentagram points indicate the goal point (see **Fig 5**). The blue crosses denote the position of the OS, and the red solid lines represent its trajectory. The green solid points and red solid points correspond to the ungrouped and grouped TSs, respectively. The black solid lines represent the vector lines of the vessels, and the black circles indicate the boundaries of the ship groups.

The results for Scenario 1 demonstrate the effectiveness of the proposed method in grouping three vessels with similar motion trends into a single cluster (see **Fig 5(a)**). By treating this cluster as a large obstacle, the OS successfully avoids it by altering its course to starboard early (see **Fig 5(c)**). In contrast, without BRB clustering, the OS passes dangerously close to two vessels, with the closest distance to each being less than 0.5 nm (see **Fig 5(d)**). This maneuver is particularly risky, as the vessels are not only in close proximity but also moving in the same direction, potentially engaged in activities like trawling, which increases the likelihood of a collision. This clear contrast underscores the superior performance of the proposed method in effectively mitigating collision risks.

It is also worth noting that, due to the quality of the AIS data, the three-vessel group (see **Fig 5(a)**) is reduced to a two-vessel group (see **Fig 5(c)** and **5(f)**). However, this reduction does not affect the overall collision avoidance performance of the method.

#### 4.3.2. Test Scenario 2.

**Fig 6:**
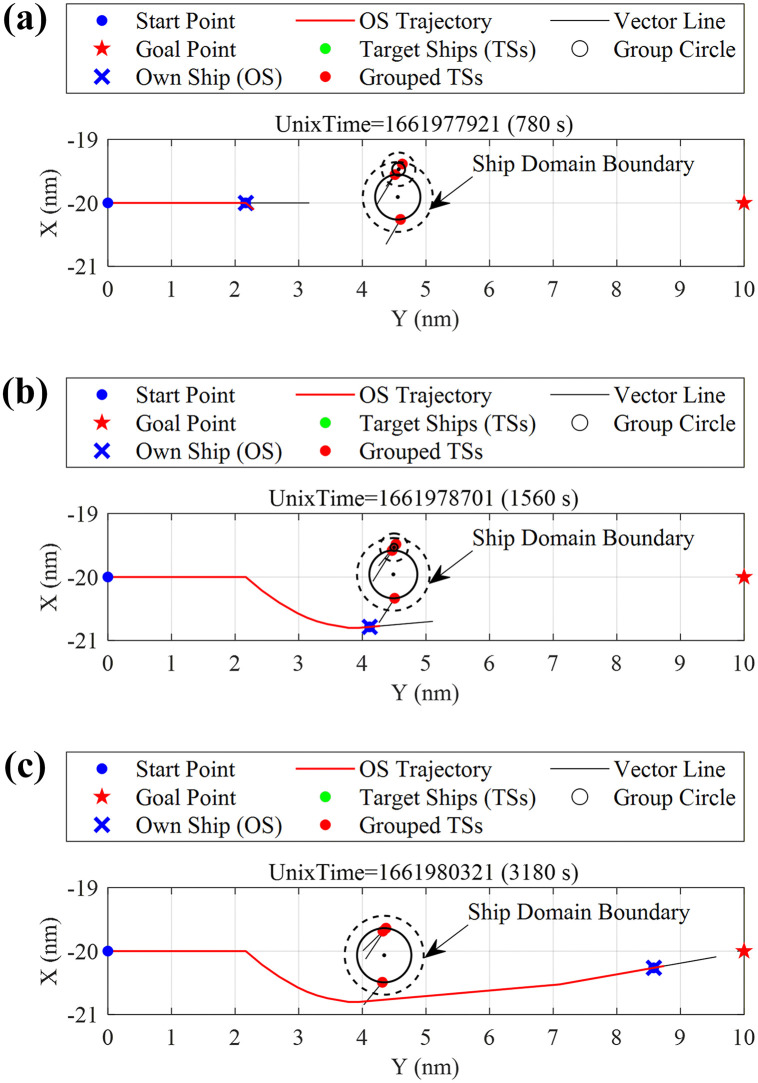
Collision avoidance path planning results for Scenario 2 with BRB clustering. Subfigures **(a)**, **(b)**, and **(c)** show the path planning process at different stages.

**Fig 7:**
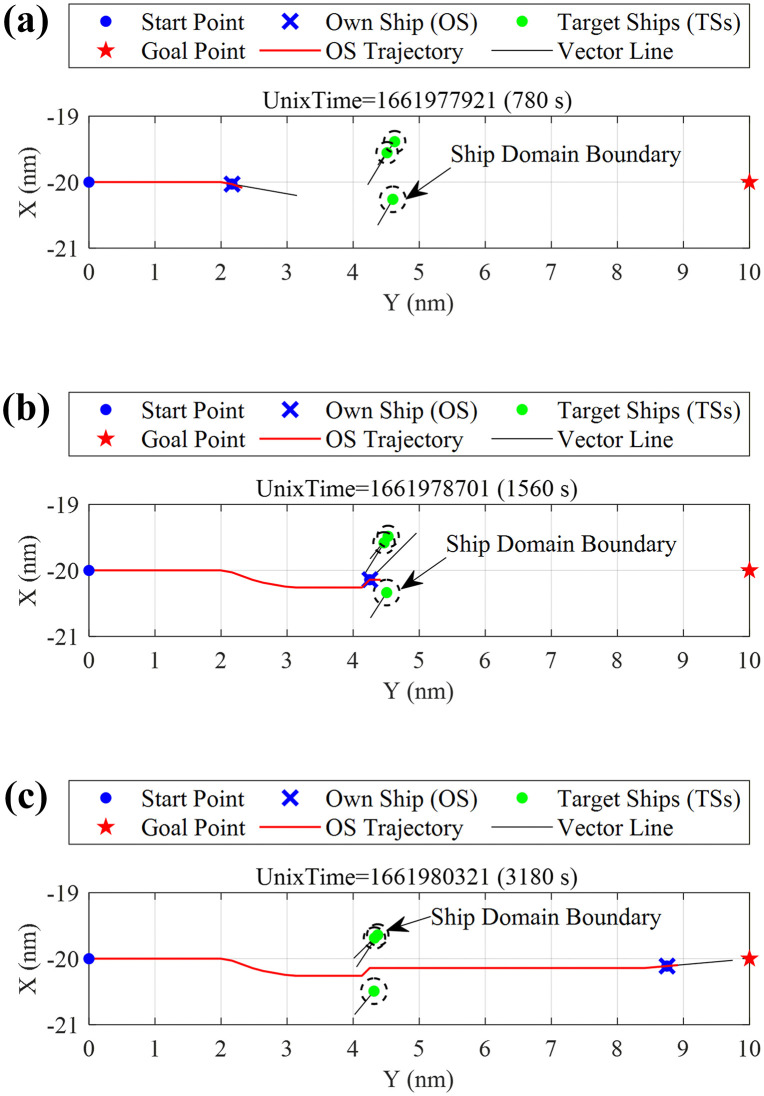
Collision avoidance path planning results for Scenario 2 without BRB clustering. Subfigures **(a)**, **(b)**, and **(c)** correspond to the same stages shown in Fig 6, allowing a direct comparison between the path planning results with and without BRB clustering.

In Test Scenario 2, the test was designed to provide a clearer and more intuitive comparison between collision avoidance path planning with and without BRB clustering. The aim was to demonstrate the difference in performance when vessels are grouped based on their motion trends, as opposed to when no grouping is applied. The goal point is set to (−20, 10) nm, with the starting position of the OS at (−20, 0) nm and an initial course of 90 degrees. **Figs 6** and **7** show the collision avoidance results. **Fig 6** shows the results with BRB clustering, while **Fig 7** shows the results without BRB clustering. This comparison helps to highlight how the grouping of vessels influences the collision avoidance process, especially in scenarios involving overlapping obstacle zones.

**Fig 6** shows that the TSs are grouped into two clusters, and their obstacle zones overlap (see **Fig 6(a)**). When the OS is about 2.5 nm away from the center of the TS group, it begins to turn to starboard for collision avoidance (see **Fig 6(b)**). As the TS group approaches the port beam of the OS, the OS adjusts its course toward the target point (see **Fig 6(c)**) and continues its navigation smoothly. By grouping the TSs based on their similar motion trends, the proposed method significantly simplifies the collision avoidance scenario, making the path planning more effective and safer.

In contrast, **Fig 7** shows the scenario without BRB clustering, where the TSs are not grouped (see Fig 7(a)). In this case, the OS adjusts its course when it is about 2.5 nm away from the TSs (see **Fig 7(b)**). However, instead of maintaining a safe distance, the OS ultimately passes through the region occupied by multiple TSs, with a minimum distance of approximately 0.5 nm between the OS and the closest vessels (see **Fig 7(c)**). This maneuver is highly risky, as any sudden change in the movement of the TSs could lead to a collision.

This test scenario effectively demonstrates the advantage of the proposed method in reducing the complexity of collision avoidance in multi-ship situations. By grouping vessels with similar motion trends, the proposed method prevents the OS from navigating through densely packed multi-ship regions. This approach not only simplifies the collision avoidance task but also ensures more efficient and safer navigation in scenarios with multiple vessels.

## 5. Conclusion

This study presents a novel integrated approach combining BRB reasoning with the VO algorithm to enhance situational awareness and optimize multi-ship collision avoidance path planning. By utilizing AIS data, the BRB reasoning framework dynamically groups ships with similar navigation trends, thereby reducing both the number of avoidance targets and the complexity of maneuvering decisions. This dynamic clustering process simplifies the path planning, ensuring more efficient and safer navigation in complex maritime environments.

The integration of the VO algorithm further enhances the approach by generating safe and feasible avoidance paths that respect real-world maritime constraints, such as vessel sizes, maneuverability, and relative velocities. The effectiveness of this method was demonstrated through scenario-based simulations, which showed that BRB-driven clustering allows the OS to treat cohesive ship groups as single obstacles. This significantly reduces the risk of crossing into regions occupied by ships with similar motion patterns, thus avoiding unnecessary conflicts and collisions. When compared to scenarios without BRB clustering, the proposed method yields safer, more efficient collision avoidance paths.

This research contributes to the advancement of intelligent maritime collision avoidance systems by providing a scalable, adaptive decision-making framework that can be integrated into future autonomous maritime operations. The results validate the potential of BRB reasoning in reducing collision risks in multi-ship environments, supporting the sustainable development of maritime transportation.

Future work will focus on improvements in both the clustering method and the collision-avoidance algorithm. For the clustering component, the emphasis will be on enhancing the BRB reasoning process by incorporating additional environmental and operational factors to improve the accuracy of ship-group identification. For the collision-avoidance algorithm, future efforts will aim to optimize the generated trajectories by considering broader factors such as energy consumption and route length, followed by full-scale validation using real vessel operations. These enhancements will further promote the transition toward fully autonomous maritime operations and improve the overall safety and efficiency of the shipping industry.
