# Supplementary material for: A path planning approach for ship collision avoidance integrating BRB reasoning and velocity obstacle algorithm
Source: PLoS One. 2026 Jun 17;21(6):e0349943. doi: 10.1371/journal.pone.0349943 (PMC13274896; doi:10.1371/journal.pone.0349943)
Supplement: Attachment — Submitted filename: Response to Reviewers.docx [file pone.0349943.s001.docx]

**Response to reviewers’ comments by authors**

**Manuscript number: PONE-D-25-48654**

**Title:** A path planning approach for ship collision avoidance integrating BRB reasoning and velocity obstacle algorithm

**Authors:** Minmin Zheng, Shuwu Wang, Haiyang Tu, Qianyu Lu

We thank PLOS ONE and the reviewers for the time and very thorough review of our paper. We have provided our answers to each reviewer’s questions as follows:

**Response to Reviewer #1 Comments**

The manuscript presents a novel and technically relevant approach that integrates Belief-Rule-Based (BRB) reasoning with the Velocity Obstacle (VO) algorithm for ship collision avoidance. The research topic is timely and of interest to the maritime and autonomous navigation community. The methodology appears logically designed, and the simulation results demonstrate the potential effectiveness of the proposed approach. However, the overall quality of writing and formatting requires significant improvement before the paper can be considered for publication.

**Point 1:** The title is too long and contains multiple technical expressions. It could be shortened or restructured for clarity and accessibility. The abstract also includes very long and complex sentences, which make it difficult to follow the main message. The authors should consider simplifying sentence structures and using clearer transitions.

**Response 1:** Thank you for this helpful comment on the paper title. We fully agree that clarity and accessibility are important.

In our work, however, the main contribution is the integration of two key techniques—BRB reasoning and the velocity obstacle algorithm—within a unified path-planning framework for ship collision avoidance. Both techniques are indispensable to the proposed method and play complementary roles. If either “BRB reasoning” or “velocity obstacle algorithm” were removed from the title, it would no longer accurately reflect the integrated nature of our approach.

For this reason, we would like to retain the original title: “*A path planning approach for ship collision avoidance integrating BRB reasoning and velocity obstacle algorithm*.”

We acknowledge that the title is somewhat technical, but we believe it remains sufficiently concise and appropriate for the journal’s specialist readership. To further improve accessibility, we have clarified the meanings of BRB reasoning and the velocity obstacle algorithm in the Abstract and Introduction.

Thank you for this helpful comment on the paper abstract. We have rewritten and abstract as follows:

“Reliable path planning for ship collision avoidance remains challenging in complex maritime environments with dense traffic. This study proposes a novel approach that integrates Belief-Rule-Based (BRB) reasoning with the Velocity Obstacle (VO) algorithm for multi-ship encounter scenarios. First, the BRB framework groups ships with similar movement trends and close proximity, reducing both the number of avoidance targets and decision-making complexity. Based on the grouping results, the safety domain radius for individual ships and ship groups is set proportionally to ship length. Subsequently, the VO algorithm generates safe and feasible avoidance trajectories toward the goal. The method was tested in two scenarios, showing improved navigational safety and operational efficiency in congested waters. This work provides a foundation for intelligent decision-making in maritime safety management and supports sustainable development in the shipping industry.“

**Point 2:** The section headings and numbering are not fully consistent across the manuscript. Please ensure uniform formatting, capitalization, and numbering according to PLOS ONE style guidelines.

**Response 2:** Thank you for this comment. The current version of the manuscript was prepared directly using the official PLOS ONE template file (“Plos_One_template.docx”), and the section headings and numbering were formatted according to the template. To address your concern, we have nonetheless carefully rechecked all headings in the revised manuscript to ensure that their formatting and capitalization are fully consistent with the PLOS ONE style guidelines.

We hope that the revised version now meets the journal’s requirements.

**Point 3:** The manuscript contains several grammatical inconsistencies, including tense shifts and awkward phrasing. Technical terms such as velocity obstacle algorithm and VO-based method are used interchangeably; a consistent terminology should be adopted throughout. A careful grammar check and professional English editing are strongly recommended.

**Response 3:** Thank you for this helpful comment. In the revised manuscript, we have carefully checked and corrected the grammar, removed tense inconsistencies, and improved awkward phrasing throughout. In addition, we have standardized the terminology related to the velocity obstacle–based technique by adopting a single, consistent term (with its abbreviation defined at first occurrence) and using it uniformly across the paper. We hope that the language and terminology are now clearer and more consistent.

**Point 4:** The in-text references to figures and tables are inconsistent. Expressions such as “Figure 3 shows…” and “As shown in Figure 3…” should follow a single formal pattern. Each figure and table should also be clearly explained within the text to guide the reader.

**Response 4:** Thank you for this useful comment. In the revised manuscript, we have carefully checked all in-text references to figures and tables and standardized the wording to follow a consistent formal pattern when referring to them. We have also ensured that each figure and table is explicitly introduced and explained in the text, so that its content and role in the argument are clear to the reader.

We hope that these revisions improve the readability and consistency of the manuscript.

**Point 5:** Some transitions between sections, particularly between the Literature Review and Proposed Approach, are abrupt. Adding short bridging or summary sentences would greatly improve the logical flow and coherence of the paper.

**Response 5:** Thank you for this helpful comment. We have revised the transitions between the relevant sections and added short bridging sentences where needed. All corresponding changes have been marked in the revised manuscript.

**Point 6:** The reference list shows inconsistencies in punctuation, capitalization, and DOI presentation. The authors should carefully check reference formatting and align it with PLOS ONE reference requirements.

**Response 6:** Thank you for this helpful comment. We have carefully checked and corrected the punctuation, capitalization, and DOI presentation in the reference list to align with the PLOS ONE reference requirements. All corresponding changes have been made in the revised manuscript.

**Response to Reviewer #2 Comments**

The manuscript entitled “A path planning approach for ship collision avoidance integrating BRB reasoning and velocity obstacle algorithm” presents a novel approach that integrates Belief-Rule-Based (BRB) reasoning with the Velocity Obstacle (VO) algorithm to address multi-ship collision avoidance scenarios. The topic is timely and relevant, and the proposed method has potential to contribute to the advancement of intelligent maritime navigation. However, several issues need to be addressed before the manuscript can be considered for publication.

**Point 1:** Original: "Zhai et al. [13] … Yu et al. [14] introduced a dynamic path planning method …"

Comment: The review is largely descriptive and lists existing studies without critical evaluation. Please strengthen the discussion by pointing out their limitations (e.g., limited real-time performance, lack of interpretability, insufficient handling of vessel clusters), thereby sharpening the motivation for the present study.

**Response 1:** Thank you for your suggestion. We have added a description of existing studies limitations to Gaps and Future Directions:

Unlike traditional clustering methods (e.g., DCA [14]) that only perform clustering or cooperative collision-avoidance approaches (e.g., GRVO [20]) that consider every ship in the scene, this study introduces a new BRB–VO integrated framework. In this framework, the BRB-based dynamic grouping reduces the decision dimensionality by clustering vessels with similar navigational trends, while the VO algorithm constructs the corresponding safety domains and generates feasible avoidance trajectories. This integration prevents the overly conservative paths that often result from static grouping and enhances adaptability in highly dynamic maritime environments.

**Point 2:** Original: "The loss function for the VO algorithm is defined as … minimizing the loss function ."

Comment: The weight parameters in the loss function are introduced without justification. A sensitivity analysis or reference to prior studies is necessary to demonstrate that the chosen values are reasonable and robust.

**Response 2:** Thank you for this insightful comment. In the revised manuscript, we have clarified the structure of the loss function and the role of the weight parameter $\beta$. The loss function is defined as the weighted average of two terms, representing the change in speed and the change in heading, respectively.

To explain the choice and effect of $\beta$, we have added the following text to the manuscript:

“A larger β increases the penalty on speed changes and thus favors solutions with nearly unchanged speed, whereas a smaller β increases the penalty on heading changes and thus favors solutions with nearly unchanged heading. In practice, β is selected according to the control preference of the navigator and the specific operational scenario.”

This addition clarifies how $\beta$balances the two indicators and how its value is determined in practical applications, thereby providing a justification for the weight parameter used in the loss function.

**Point 3:** Original: "In coastal waters, one of the key challenges is the dense congregation of fishing vessels … inadequate."

Comment: This could be condensed. At the same time, consider adding concrete statistics (e.g., vessel density, fishing fleet size) to strengthen the motivation.

**Response 3:** Thank you for this helpful comment. In the revised manuscript, we have condensed the description of the coastal fishing scenario to make the motivation more concise and focused. The paragraph now emphasizes the dense concentration of trawl fishing vessels, their similar movement patterns, and the resulting limitations of conventional collision-avoidance methods.

Regarding concrete statistics, detailed quantitative data (e.g., vessel density and fleet size) for the specific waters considered in this study are not readily available from public sources. Therefore, instead of providing potentially inaccurate numerical estimates, we have strengthened the qualitative description of the operating characteristics of trawl fishing fleets and their impact on collision-avoidance performance, supported by the existing reference [3]. We hope that this revision clarifies the motivation while remaining faithful to the available data.

**Point 4:** Original: "Metric x4: Average Speed, defined as follows …"

Comment: The choice of average speed as a clustering metric requires further justification. Please elaborate on its physical relevance and limitations in dynamic environments.

**Response 4:** Thank you for this helpful comment. In the revised manuscript, we have clarified the physical meaning of the clustering metrics $x_{1}$–$x_{5}$. Specifically, $x_{1}$(Distance) describes the spatial proximity between the two vessels; $x_{2}$(Speed Difference) and $x_{3}$(Course Difference) jointly describe how similar their motion trends are; $x_{4}$(Average Speed) reflects the overall speed level of the two vessels in the encounter scenario; and $x_{5}$(Course Bearing Angle) describes their relative attitude, i.e., the geometric orientation of one vessel with respect to the other. We believe that this additional explanation makes the rationale and physical interpretation of the selected metrics clearer.

**Point 5:** The method is tested on limited AIS data from 2019 in a regional case. It is not clear how the algorithm scales to high-density international traffic (hundreds of ships). Please at least discuss computational complexity.

**Response 5:** Thank you for this important comment. In the revised manuscript, we have added a discussion on the computational complexity and scalability of the proposed method. Specifically, we clarify that the algorithm is implemented in a local manner: for each own ship, only vessels within a predefined interaction radius are considered as potential encounter partners. Let $K$denote the number of such neighboring vessels. For each planning step, the main computational cost scales approximately linearly with $K$, rather than with the total number of ships in the wider region. In high-density traffic, although the total number of ships may reach the order of hundreds, the number of effective neighbors around any given ship remains limited by the chosen interaction radius, which bounds the computational load. We hope that this additional explanation addresses the reviewer’s concern about scalability.

**Point 6:** The current future work statement (L785–L791) is vague. More concrete directions should be suggested, such as integration with reinforcement learning, testing with real-time ship simulators, or hybridization with predictive models.

**Response 6:** Thank you for your suggestion. We have added more concrete directions into artiacl:

Future work will focus on improvements in both the clustering method and the collision-avoidance algorithm. For the clustering component, the emphasis will be on enhancing the BRB reasoning process by incorporating additional environmental and operational factors to improve the accuracy of ship-group identification. For the collision-avoidance algorithm, future efforts will aim to optimize the generated trajectories by considering broader factors such as energy consumption and route length, followed by full-scale validation using real vessel operations. These enhancements will further promote the transition toward fully autonomous maritime operations and improve the overall safety and efficiency of the shipping industry.

**Point 7:** Sentences are sometimes too long and contain multiple clauses (e.g., L25–L31 in the introduction). Shorter and more precise sentences would improve readability. A language polish is recommended.

**Response 7:** Thank you for this helpful comment. We have carefully checked the entire manuscript, with particular attention to the long sentences mentioned (e.g., L25–L31 in the introduction), and have revised them into shorter and more precise sentences to improve readability. A general language polish has also been carried out throughout the paper.
