# Supplementary material for: A path planning approach for ship collision avoidance integrating BRB reasoning and velocity obstacle algorithm
Source: PLoS One. 2026 Jun 17;21(6):e0349943. doi: 10.1371/journal.pone.0349943 (PMC13274896; doi:10.1371/journal.pone.0349943)
Supplement: Peer review history

# Peer review: Decision Letter 0

**DOI:** 10.1371/journal.pone.0349943.r001

**Authors:** Yongxiang Zhang

11 Nov 2025

PONE-D-25-48654

A path planning approach for ship collision avoidance integrating BRB reasoning and velocity obstacle algorithm

PLOS ONE

Dear Dr. Zheng,

Thank you for submitting your manuscript to PLOS ONE. After careful consideration, we feel that it has merit but does not fully meet PLOS ONE’s publication criteria as it currently stands. Therefore, we invite you to submit a revised version of the manuscript that addresses the points raised during the review process.

Please submit your revised manuscript by Dec 26 2025 11:59PM. If you will need more time than this to complete your revisions, please reply to this message or contact the journal office at plosone@plos.org. When you're ready to submit your revision, log on to https://www.editorialmanager.com/pone/ and select the 'Submissions Needing Revision' folder to locate your manuscript file.

Please include the following items when submitting your revised manuscript:

- A rebuttal letter that responds to each point raised by the academic editor and reviewer(s). You should upload this letter as a separate file labeled 'Response to Reviewers'.
- A marked-up copy of your manuscript that highlights changes made to the original version. You should upload this as a separate file labeled 'Revised Manuscript with Track Changes'.
- An unmarked version of your revised paper without tracked changes. You should upload this as a separate file labeled 'Manuscript'.

If you would like to make changes to your financial disclosure, please include your updated statement in your cover letter. Guidelines for resubmitting your figure files are available below the reviewer comments at the end of this letter.

If applicable, we recommend that you deposit your laboratory protocols in protocols.io to enhance the reproducibility of your results. Protocols.io assigns your protocol its own identifier (DOI) so that it can be cited independently in the future. For instructions see: https://journals.plos.org/plosone/s/submission-guidelines#loc-laboratory-protocols. Additionally, PLOS ONE offers an option for publishing peer-reviewed Lab Protocol articles, which describe protocols hosted on protocols.io. Read more information on sharing protocols at https://plos.org/protocols?utm_medium=editorial-email&utm_source=authorletters&utm_campaign=protocols.

We look forward to receiving your revised manuscript.

Kind regards,

Yongxiang Zhang, Ph.D.

Academic Editor

PLOS ONE

Journal Requirements:

When submitting your revision, we need you to address these additional requirements.

1. Please ensure that your manuscript meets PLOS ONE's style requirements, including those for file naming. The PLOS ONE style templates can be found at

https://journals.plos.org/plosone/s/file?id=wjVg/PLOSOne_formatting_sample_main_body.pdf and

https://journals.plos.org/plosone/s/file?id=ba62/PLOSOne_formatting_sample_title_authors_affiliations.pdf

2. Thank you for stating the following in the Competing Interests/Financial Disclosure section:

The authors have declared that no competing interests exist.

We note that one or more of the authors are employed by a commercial company: Shanghai Ship and Shipping Research Institute Co.,Ltd.

a. Please provide an amended Funding Statement declaring this commercial affiliation, as well as a statement regarding the Role of Funders in your study. If the funding organization did not play a role in the study design, data collection and analysis, decision to publish, or preparation of the manuscript and only provided financial support in the form of authors' salaries and/or research materials, please review your statements relating to the author contributions, and ensure you have specifically and accurately indicated the role(s) that these authors had in your study. You can update author roles in the Author Contributions section of the online submission form.

Please also include the following statement within your amended Funding Statement.

“The funder provided support in the form of salaries for authors [insert relevant initials], but did not have any additional role in the study design, data collection and analysis, decision to publish, or preparation of the manuscript. The specific roles of these authors are articulated in the ‘author contributions’ section.”

If your commercial affiliation did play a role in your study, please state and explain this role within your updated Funding Statement.

b. Please also provide an updated Competing Interests Statement declaring this commercial affiliation along with any other relevant declarations relating to employment, consultancy, patents, products in development, or marketed products, etc.

Within your Competing Interests Statement, please confirm that this commercial affiliation does not alter your adherence to all PLOS ONE policies on sharing data and materials by including the following statement: "This does not alter our adherence to PLOS ONE policies on sharing data and materials.” (as detailed online in our guide for authors http://journals.plos.org/plosone/s/competing-interests) . If this adherence statement is not accurate and there are restrictions on sharing of data and/or materials, please state these. Please note that we cannot proceed with consideration of your article until this information has been declared.

Please include both an updated Funding Statement and Competing Interests Statement in your cover letter. We will change the online submission form on your behalf.

3. We note that Figure 3 in your submission contain map images which may be copyrighted. All PLOS content is published under the Creative Commons Attribution License (CC BY 4.0), which means that the manuscript, images, and Supporting Information files will be freely available online, and any third party is permitted to access, download, copy, distribute, and use these materials in any way, even commercially, with proper attribution. For these reasons, we cannot publish previously copyrighted maps or satellite images created using proprietary data, such as Google software (Google Maps, Street View, and Earth). For more information, see our copyright guidelines: http://journals.plos.org/plosone/s/licenses-and-copyright.

We require you to either (a) present written permission from the copyright holder to publish these figures specifically under the CC BY 4.0 license, or (b) remove the figures from your submission:

a. You may seek permission from the original copyright holder of Figure 3 to publish the content specifically under the CC BY 4.0 license.

We recommend that you contact the original copyright holder with the Content Permission Form (http://journals.plos.org/plosone/s/file?id=7c09/content-permission-form.pdf) and the following text:

“I request permission for the open-access journal PLOS ONE to publish XXX under the Creative Commons Attribution License (CCAL) CC BY 4.0 (http://creativecommons.org/licenses/by/4.0/). Please be aware that this license allows unrestricted use and distribution, even commercially, by third parties. Please reply and provide explicit written permission to publish XXX under a CC BY license and complete the attached form.”

Please upload the completed Content Permission Form or other proof of granted permissions as an ""Other"" file with your submission.

In the figure caption of the copyrighted figure, please include the following text: “Reprinted from [ref] under a CC BY license, with permission from [name of publisher], original copyright [original copyright year].”

b. If you are unable to obtain permission from the original copyright holder to publish these figures under the CC BY 4.0 license or if the copyright holder’s requirements are incompatible with the CC BY 4.0 license, please either i) remove the figure or ii) supply a replacement figure that complies with the CC BY 4.0 license. Please check copyright information on all replacement figures and update the figure caption with source information. If applicable, please specify in the figure caption text when a figure is similar but not identical to the original image and is therefore for illustrative purposes only.

The following resources for replacing copyrighted map figures may be helpful:

USGS National Map Viewer (public domain): http://viewer.nationalmap.gov/viewer/

The Gateway to Astronaut Photography of Earth (public domain): http://eol.jsc.nasa.gov/sseop/clickmap/

Maps at the CIA (public domain): https://www.cia.gov/library/publications/the-world-factbook/index.html and https://www.cia.gov/library/publications/cia-maps-publications/index.html

NASA Earth Observatory (public domain): http://earthobservatory.nasa.gov/

Landsat: http://landsat.visibleearth.nasa.gov/

USGS EROS (Earth Resources Observatory and Science (EROS) Center) (public domain): http://eros.usgs.gov/#

Natural Earth (public domain): http://www.naturalearthdata.com/

4. If the reviewer comments include a recommendation to cite specific previously published works, please review and evaluate these publications to determine whether they are relevant and should be cited. There is no requirement to cite these works unless the editor has indicated otherwise.

[Note: HTML markup is below. Please do not edit.]

Reviewers' comments:

Reviewer's Responses to Questions

**Comments to the Author**

1. Is the manuscript technically sound, and do the data support the conclusions?

The manuscript must describe a technically sound piece of scientific research with data that supports the conclusions. Experiments must have been conducted rigorously, with appropriate controls, replication, and sample sizes. The conclusions must be drawn appropriately based on the data presented.

Reviewer #1: Yes

Reviewer #2: Yes

**********

2. Has the statistical analysis been performed appropriately and rigorously?

Reviewer #1: I Don't Know

Reviewer #2: Yes

**********

3. Have the authors made all data underlying the findings in their manuscript fully available?

The PLOS Data policy requires authors to make all data underlying the findings described in their manuscript fully available without restriction, with rare exception (please refer to the Data Availability Statement in the manuscript PDF file). The data should be provided as part of the manuscript or its supporting information, or deposited to a public repository. For example, in addition to summary statistics, the data points behind means, medians and variance measures should be available. If there are restrictions on publicly sharing data—e.g. participant privacy or use of data from a third party—those must be specified.

Reviewer #1: No

Reviewer #2: Yes

**********

4. Is the manuscript presented in an intelligible fashion and written in standard English?

PLOS ONE does not copyedit accepted manuscripts, so the language in submitted articles must be clear, correct, and unambiguous. Any typographical or grammatical errors should be corrected at revision, so please note any specific errors here.

Reviewer #1: Yes

Reviewer #2: Yes

**********

5. Review Comments to the Author

Please use the space provided to explain your answers to the questions above. You may also include additional comments for the author, including concerns about dual publication, research ethics, or publication ethics. (Please upload your review as an attachment if it exceeds 20,000 characters)

Reviewer #1: The manuscript presents a novel and technically relevant approach that integrates Belief-Rule-Based (BRB) reasoning with the Velocity Obstacle (VO) algorithm for ship collision avoidance. The research topic is timely and of interest to the maritime and autonomous navigation community. The methodology appears logically designed, and the simulation results demonstrate the potential effectiveness of the proposed approach. However, the overall quality of writing and formatting requires significant improvement before the paper can be considered for publication.

• The title is too long and contains multiple technical expressions. It could be shortened or restructured for clarity and accessibility. The abstract also includes very long and complex sentences, which make it difficult to follow the main message. The authors should consider simplifying sentence structures and using clearer transitions.

• The section headings and numbering are not fully consistent across the manuscript. Please ensure uniform formatting, capitalization, and numbering according to PLOS ONE style guidelines.

• The manuscript contains several grammatical inconsistencies, including tense shifts and awkward phrasing. Technical terms such as velocity obstacle algorithm and VO-based method are used interchangeably; a consistent terminology should be adopted throughout. A careful grammar check and professional English editing are strongly recommended.

• The in-text references to figures and tables are inconsistent. Expressions such as “Figure 3 shows…” and “As shown in Figure 3…” should follow a single formal pattern. Each figure and table should also be clearly explained within the text to guide the reader.

• Some transitions between sections, particularly between the Literature Review and Proposed Approach, are abrupt. Adding short bridging or summary sentences would greatly improve the logical flow and coherence of the paper.

• The reference list shows inconsistencies in punctuation, capitalization, and DOI presentation. The authors should carefully check reference formatting and align it with PLOS ONE reference requirements.

Reviewer #2: The manuscript entitled “A path planning approach for ship collision avoidance integrating BRB reasoning and velocity obstacle algorithm” presents a novel approach that integrates Belief-Rule-Based (BRB) reasoning with the Velocity Obstacle (VO) algorithm to address multi-ship collision avoidance scenarios. The topic is timely and relevant, and the proposed method has potential to contribute to the advancement of intelligent maritime navigation. However, several issues need to be addressed before the manuscript can be considered for publication.

1. Original: "Zhai et al. [13] … Yu et al. [14] introduced a dynamic path planning method …"

Comment: The review is largely descriptive and lists existing studies without critical evaluation. Please strengthen the discussion by pointing out their limitations (e.g., limited real-time performance, lack of interpretability, insufficient handling of vessel clusters), thereby sharpening the motivation for the present study.

2. Original: "The loss function for the VO algorithm is defined as … minimizing the loss function ."

Comment: The weight parameters in the loss function are introduced without justification. A sensitivity analysis or reference to prior studies is necessary to demonstrate that the chosen values are reasonable and robust.

3. Original: "In coastal waters, one of the key challenges is the dense congregation of fishing vessels … inadequate."

Comment: This could be condensed. At the same time, consider adding concrete statistics (e.g., vessel density, fishing fleet size) to strengthen the motivation.

4. Original: "Metric x4: Average Speed, defined as follows …"

Comment: The choice of average speed as a clustering metric requires further justification. Please elaborate on its physical relevance and limitations in dynamic environments.

5. The method is tested on limited AIS data from 2019 in a regional case. It is not clear how the algorithm scales to high-density international traffic (hundreds of ships). Please at least discuss computational complexity.

6. The current future work statement (L785–L791) is vague. More concrete directions should be suggested, such as integration with reinforcement learning, testing with real-time ship simulators, or hybridization with predictive models.

7. Sentences are sometimes too long and contain multiple clauses (e.g., L25–L31 in the introduction). Shorter and more precise sentences would improve readability. A language polish is recommended.

**********

6. PLOS authors have the option to publish the peer review history of their article (what does this mean?). If published, this will include your full peer review and any attached files.

If you choose “no”, your identity will remain anonymous but your review may still be made public.

**Do you want your identity to be public for this peer review?**   For information about this choice, including consent withdrawal, please see our Privacy Policy.

Reviewer #1: No

Reviewer #2: No

**********

[NOTE: If reviewer comments were submitted as an attachment file, they will be attached to this email and accessible via the submission site. Please log into your account, locate the manuscript record, and check for the action link "View Attachments". If this link does not appear, there are no attachment files.]

To ensure your figures meet our technical requirements, please review our figure guidelines: https://journals.plos.org/plosone/s/figures

You may also use PLOS’s free figure tool, NAAS, to help you prepare publication quality figures: https://journals.plos.org/plosone/s/figures#loc-tools-for-figure-preparation.

NAAS will assess whether your figures meet our technical requirements by comparing each figure against our figure specifications.

# Author comment: Author response to Decision Letter 1

**DOI:** 10.1371/journal.pone.0349943.r002

24 Dec 2025

We would like to express our sincere gratitude to PLOS ONE and the two reviewers for their meticulous and constructive comments on our manuscript (Manuscript number: PONE-D-25-48654). We have carefully considered each comment and addressed all the issues raised in detail in the attached point-by-point responses.

In the revised manuscript, key improvements include: optimizing the abstract for clarity and readability while retaining the original title (to accurately reflect the integrated nature of our approach); standardizing section headings, numbering, terminology, and in-text references to figures/tables in strict accordance with PLOS ONE style guidelines; correcting grammatical inconsistencies and refining awkward phrasing through comprehensive language polishing; enhancing logical coherence by adding bridging sentences between relevant sections (e.g., Literature Review and Proposed Approach); revising the reference list to ensure consistency in punctuation, capitalization, and DOI presentation; supplementing critical content such as limitations of existing studies, justification for weight parameters in the loss function, physical interpretation of clustering metrics, and a detailed discussion on computational complexity and scalability; condensing redundant descriptions and strengthening the motivation for the study; and specifying concrete directions for future work.

All revisions have been clearly marked in the revised manuscript. We believe these changes have significantly improved the clarity, consistency, scientific rigor, and readability of the paper. We sincerely hope that the revised version meets the journal’s publication standards and kindly request the editors and reviewers to consider it for publication. Thank you again for your valuable time and guidance.

Attachment Submitted filename: Response to Reviewers.docx

# Peer review: Decision Letter 1

**DOI:** 10.1371/journal.pone.0349943.r003

**Authors:** Jiapeng Liu

15 Apr 2026

PONE-D-25-48654R1

A path planning approach for ship collision avoidance integrating BRB reasoning and velocity obstacle algorithm

PLOS One

Dear Dr. Zheng,

Thank you for submitting your manuscript to PLOS ONE. After careful consideration, we feel that it has merit but does not fully meet PLOS ONE’s publication criteria as it currently stands. Therefore, we invite you to submit a revised version of the manuscript that addresses the points raised during the review process.

The readability of this paper needs to be further improved.

Please submit your revised manuscript by May 30 2026 11:59PM. If you will need more time than this to complete your revisions, please reply to this message or contact the journal office at plosone@plos.org. When you're ready to submit your revision, log on to https://www.editorialmanager.com/pone/ and select the 'Submissions Needing Revision' folder to locate your manuscript file.

Please include the following items when submitting your revised manuscript:

- A letter that responds to each point raised by the academic editor and reviewer(s). You should upload this letter as a separate file labeled 'Response to Reviewers'.
- A marked-up copy of your manuscript that highlights changes made to the original version. You should upload this as a separate file labeled 'Revised Manuscript with Track Changes'.
- An unmarked version of your revised paper without tracked changes. You should upload this as a separate file labeled 'Manuscript'.

If you would like to make changes to your financial disclosure, please include your updated statement in your cover letter. Guidelines for resubmitting your figure files are available below the reviewer comments at the end of this letter.

If applicable, we recommend that you deposit your laboratory protocols in protocols.io to enhance the reproducibility of your results. Protocols.io assigns your protocol its own identifier (DOI) so that it can be cited independently in the future. For instructions see: https://journals.plos.org/plosone/s/submission-guidelines#loc-laboratory-protocols. Additionally, PLOS ONE offers an option for publishing peer-reviewed Lab Protocol articles, which describe protocols hosted on protocols.io. Read more information on sharing protocols at https://plos.org/protocols?utm_medium=editorial-email&utm_source=authorletters&utm_campaign=protocols.

As the corresponding author, your ORCID iD is verified in the submission system and will appear in the published article. PLOS supports the use of ORCID, and we encourage all coauthors to register for an ORCID iD and use it as well. Please encourage your coauthors to verify their ORCID iD within the submission system before final acceptance, as unverified ORCID iDs will not appear in the published article. *Only*   the individual author can complete the verification step; PLOS staff *cannot*   verify ORCID iDs on behalf of authors.

We look forward to receiving your revised manuscript.

Kind regards,

Jiapeng Liu, Ph.D.

Academic Editor

PLOS One

Journal Requirements:

If the reviewer comments include a recommendation to cite specific previously published works, please review and evaluate these publications to determine whether they are relevant and should be cited. There is no requirement to cite these works unless the editor has indicated otherwise.

Please review your reference list to ensure that it is complete and correct. If you have cited papers that have been retracted, please include the rationale for doing so in the manuscript text, or remove these references and replace them with relevant current references. Any changes to the reference list should be mentioned in the rebuttal letter that accompanies your revised manuscript. If you need to cite a retracted article, indicate the article’s retracted status in the References list and also include a citation and full reference for the retraction notice.

Additional Editor Comments:

The readability of this paper needs to be further improved, with specific comments as follows.

1. The first-level and second-level headings of the paper need to be reorganized. The current structure results in very poor readability, which reduces the overall quality of the paper.

2. The analysis of the research status is too superficial. Please supplement the research achievements of the past three years and conduct relevant analyses.

3. The references are too outdated.

[Note: HTML markup is below. Please do not edit.]

Reviewers' comments:

Reviewer's Responses to Questions

**Comments to the Author**

1. If the authors have adequately addressed your comments raised in a previous round of review and you feel that this manuscript is now acceptable for publication, you may indicate that here to bypass the “Comments to the Author” section, enter your conflict of interest statement in the “Confidential to Editor” section, and submit your "Accept" recommendation.

Reviewer #1: All comments have been addressed

Reviewer #2: All comments have been addressed

**********

2. Is the manuscript technically sound, and do the data support the conclusions?

The manuscript must describe a technically sound piece of scientific research with data that supports the conclusions. Experiments must have been conducted rigorously, with appropriate controls, replication, and sample sizes. The conclusions must be drawn appropriately based on the data presented.

Reviewer #1: Yes

Reviewer #2: No

**********

3. Has the statistical analysis been performed appropriately and rigorously?

Reviewer #1: N/A

Reviewer #2: Yes

**********

4. Have the authors made all data underlying the findings in their manuscript fully available?

The PLOS Data policy requires authors to make all data underlying the findings described in their manuscript fully available without restriction, with rare exception (please refer to the Data Availability Statement in the manuscript PDF file). The data should be provided as part of the manuscript or its supporting information, or deposited to a public repository. For example, in addition to summary statistics, the data points behind means, medians and variance measures should be available. If there are restrictions on publicly sharing data—e.g. participant privacy or use of data from a third party—those must be specified.

Reviewer #1: Yes

Reviewer #2: No

**********

5. Is the manuscript presented in an intelligible fashion and written in standard English?

PLOS ONE does not copyedit accepted manuscripts, so the language in submitted articles must be clear, correct, and unambiguous. Any typographical or grammatical errors should be corrected at revision, so please note any specific errors here.

Reviewer #1: Yes

Reviewer #2: Yes

**********

6. Review Comments to the Author

Please use the space provided to explain your answers to the questions above. You may also include additional comments for the author, including concerns about dual publication, research ethics, or publication ethics. (Please upload your review as an attachment if it exceeds 20,000 characters)

Reviewer #1: No further comments. All the comments have been addressed.No further comments. All the comments have been addressed.

Reviewer #2: (No Response)

**********

7. PLOS authors have the option to publish the peer review history of their article (what does this mean?). If published, this will include your full peer review and any attached files.

If you choose “no”, your identity will remain anonymous but your review may still be made public.

**Do you want your identity to be public for this peer review?**   For information about this choice, including consent withdrawal, please see our Privacy Policy.

Reviewer #1: No

Reviewer #2: No

**********

[NOTE: If reviewer comments were submitted as an attachment file, they will be attached to this email and accessible via the submission site. Please log into your account, locate the manuscript record, and check for the action link "View Attachments". If this link does not appear, there are no attachment files.]

To ensure your figures meet our technical requirements, please review our figure guidelines: https://journals.plos.org/plosone/s/figures

You may also use PLOS’s free figure tool, NAAS, to help you prepare publication quality figures: https://journals.plos.org/plosone/s/figures#loc-tools-for-figure-preparation.

NAAS will assess whether your figures meet our technical requirements by comparing each figure against our figure specifications.

# Author comment: Author response to Decision Letter 2

**DOI:** 10.1371/journal.pone.0349943.r004

5 May 2026

Dear Academic Editor and Reviewers,

Thank you for your constructive comments on our manuscript. According to the latest revision request, we have made targeted revisions to improve the readability of the manuscript. In the revised version, we reorganized the section hierarchy, strengthened the analysis of recent research progress, and increased the proportion of recent references by replacing older or less directly relevant references with 2025–2026 studies.

Response to the Academic Editor

Comment 1. The first-level and second-level headings of the paper need to be reorganized. The current structure results in very poor readability, which reduces the overall quality of the paper.

Response: Thank you for this helpful suggestion. We have reorganized the heading hierarchy of the manuscript to improve readability and logical flow. Specifically, we revised the main structure to include clearer first-level and second-level headings, such as “Research Status and Gap Analysis”, “A Proposed Approach”, “Overall Workflow of the Approach”, “Case Study and Results”, and “Test Scenarios and Comparative Results”. We also adjusted several methodological subsections, including “Selection of Clustering Metric”, “BRB Reasoning Method”, “Determination of Ship Group”, “Domain Radius of a Single Ship”, “Domain Radius of Ship Group”, and “Test Scenario 1/2”, as lower-level headings. These changes make the structure more coherent and easier to follow.

Comment 2. The analysis of the research status is too superficial. Please supplement the research achievements of the past three years and conduct relevant analyses.

Response: Thank you for this comment. We have supplemented the literature review in a targeted manner. The revised manuscript now provides a clearer summary and analysis of recent research progress in multi-ship interaction modelling, COLREGs-aware decision-making, learning-based cooperative avoidance, dynamic risk assessment, and velocity-obstacle-related collaborative collision avoidance. These additions strengthen the discussion of recent developments while maintaining the original scope and conciseness of the manuscript.

Comment 3. The references are too outdated.

Response: Thank you for this suggestion. To address this issue, we replaced several older references with recent studies published in 2025 and 2026. The revised manuscript now contains eight references from 2025–2026, including references [13], [14], [16], [20], [22], [23], [24], and [25]. These updated references better reflect recent research progress in ship collision avoidance, multi-ship encounter decision-making, COLREGs-compliant avoidance, and related computational methods.

Additional Revision: Data Availability

Response: In view of the reviewer form indicating a concern about data availability, we added a concise Data Availability Statement to the revised manuscript. The statement clarifies that the processed data supporting the simulation results and the code used in this study are available at https://figshare.com/s/8e80001a7a7a214dd6e0, while the raw AIS data used to construct the case scenarios are subject to third-party data-use restrictions and cannot be publicly shared.

We sincerely appreciate the Academic Editor’s and reviewers’ time and constructive comments. We hope that the revised manuscript now better meets the readability and reference-update requirements of PLOS ONE.

Sincerely,

Minmin Zheng and co-authors

Attachment Submitted filename: Response_to_Reviewers-260505.docx

# Editor report: Decision Letter 2

**DOI:** 10.1371/journal.pone.0349943.r005

**Authors:** Jiapeng Liu

7 May 2026

A path planning approach for ship collision avoidance integrating BRB reasoning and velocity obstacle algorithm

PONE-D-25-48654R2

Dear Dr. Zheng,

We’re pleased to inform you that your manuscript has been judged scientifically suitable for publication and will be formally accepted for publication once it meets all outstanding technical requirements.

Within one week, you’ll receive an e-mail detailing the required amendments. When these have been addressed, you’ll receive a formal acceptance letter and your manuscript will be scheduled for publication.

An invoice will be generated when your article is formally accepted. Please note, if your institution has a publishing partnership with PLOS and your article meets the relevant criteria, all or part of your publication costs will be covered. Please make sure your user information is up-to-date by logging into Editorial Manager at Editorial Manager® and clicking the ‘Update My Information' link at the top of the page. For questions related to billing, please contact billing support.

If your institution or institutions have a press office, please notify them about your upcoming paper to help maximize its impact. If they’ll be preparing press materials, please inform our press team as soon as possible -- no later than 48 hours after receiving the formal acceptance. Your manuscript will remain under strict press embargo until 2 pm Eastern Time on the date of publication. For more information, please contact onepress@plos.org.

Kind regards,

Jiapeng Liu, Ph.D.

Academic Editor

PLOS One

Additional Editor Comments (optional):

Reviewers' comments:

# Editor report: Acceptance letter

**DOI:** 10.1371/journal.pone.0349943.r006

**Authors:** Jiapeng Liu

PONE-D-25-48654R2

PLOS One

Dear Dr. Zheng,

I'm pleased to inform you that your manuscript has been deemed suitable for publication in PLOS One. Congratulations! Your manuscript is now being handed over to our production team.

At this stage, our production department will prepare your paper for publication. This includes ensuring the following:

* All references, tables, and figures are properly cited

* All relevant supporting information is included in the manuscript submission,

* There are no issues that prevent the paper from being properly typeset

You will receive further instructions from the production team, including instructions on how to review your proof when it is ready. Please keep in mind that we are working through a large volume of accepted articles, so please give us a few days to review your paper and let you know the next and final steps.

Lastly, if your institution or institutions have a press office, please let them know about your upcoming paper now to help maximize its impact. If they'll be preparing press materials, please inform our press team within the next 48 hours. Your manuscript will remain under strict press embargo until 2 pm Eastern Time on the date of publication. For more information, please contact onepress@plos.org.

You will receive an invoice from PLOS for your publication fee after your manuscript has reached the completed accept phase. If you receive an email requesting payment before acceptance or for any other service, this may be a phishing scheme. Learn how to identify phishing emails and protect your accounts at https://explore.plos.org/phishing.

If we can help with anything else, please email us at customercare@plos.org.

Thank you for submitting your work to PLOS ONE and supporting open access.

Kind regards,

PLOS ONE Editorial Office Staff

on behalf of

Dr. Jiapeng Liu

Academic Editor

PLOS One
